# Renal Impairment in Chronic Hepatitis B: A Review

**DOI:** 10.3390/diseases6020052

**Published:** 2018-06-19

**Authors:** Hiroteru Kamimura, Toru Setsu, Naruhiro Kimura, Takeshi Yokoo, Akira Sakamaki, Kenya Kamimura, Atsunori Tsuchiya, Masaaki Takamura, Satoshi Yamagiwa, Shuji Terai

**Affiliations:** Division of Gastroenterology and Hepatology, Niigata University Graduate School of Medical and Dental Sciences, Niigata 951-8510, Japan; sevendaysinthesun@icloud.com (T.S.); naruhiro-kimura@live.jp (N.K.); t-yokoo@med.niigata-u.ac.jp (T.Y.); saka-a@med.niigata-u.ac.jp (A.S.); kenya-k@med.niigata-u.ac.jp (K.K.); atsunori@med.niigata-u.ac.jp (A.T.); atmc@med.niigata-u.ac.jp (M.T.); hiroterukg@gmail.com (S.Y.); terais@med.niigata-u.ac.jp (S.T.)

**Keywords:** hepatitis B, hepatorenal syndrome, HBV-associated glomerulonephritis

## Abstract

The liver plays a key role in the metabolism of proteins. Liver dysfunction affects many organs because it communicates with the spleen and all digestive organs through the portal vein. Additionally, the kidney is an organ that is closely related to the liver and is involved in liver diseases. Glomerulonephritis is an important extrahepatic manifestation of chronic hepatitis B virus (HBV) infection. Nucleos(t)ide analog (NA) therapy effectively suppresses HBV replication by inhibiting HBV polymerase, thus decreasing the levels of serum HBV-DNA and delaying the progression of cirrhosis. Although NA therapy is recommended for all patients with chronic HBV infection, regardless of the level of renal dysfunction, there is limited information on NA use in patients with chronic kidney disease. In addition, in patients with end-stage liver cirrhosis, hepatorenal syndrome can be fatal. Hence, we should take into account the stage of impaired renal function in patients with cirrhosis. The aims of this article are to review the epidemiology, clinical presentation, treatment, and prevention of HBV-associated nephropathy.

## 1. Introduction

Despite the excellent efficacy of direct-acting antivirals (DAA) reported in hepatitis C virus (HCV), hepatitis B virus (HBV) still has problems that need to be addressed. In 1971, Combes reported on patients with hepatitis B with membranous kidney disease who presented with nephrotic syndrome. They first reported the case of a middle-aged man with membranous glomerulonephritis due to glomerular deposition of Australian-antigen-containing immune complexes [1].

Oral nucleoside/nucleotide analogs (NAs) are currently the mainstream treatment for patients with chronic hepatitis B infection. Use of NAs in HBV is generally safe, but adverse effects, such as nephrotoxicity, have been discussed [2,3]. Moreover, regarding the stage of liver cirrhosis, we should be aware of the possibility of impaired renal function at this stage of liver cirrhosis. Several biomarkers can be used to measure the early stage of impaired renal function in patients with cirrhosis. The aims of this article are to review the epidemiology, clinical presentation, treatment, and prevention of HBV-associated nephropathy.

## 2. HBV-Associated Nephropathy

Renal diseases resulting from HBV infection are membranous nephropathy (MN), membranoproliferative glomerulonephritis (MPGN), polyarteritis nodosa (PAN), and mesangial proliferative glomerulonephritis (MesPGN). The pathogenetic role of HBV infection has been documented by the demonstration of hepatitis B antigen-antibody complexes in renal lesions via immunofluorescence microscopy [4].

Glomerular disease is more common in children than in adults and in men than in women [5]. MN is the most common type of glomerulonephritis (GN) in humans and is particularly frequent in male children. By contrast, MPGN is well known in adults [6].

### 2.1. Pathology of HBV-Associated Nephropathy

#### 2.1.1. Membranous Nephropathy (MN)

Takekoshi et al. reported that HBeAg exists in the serum of cases in two different forms, free ‘small’ HBeAg and IgG-associated ‘large’ HBeAg, which is an immune complex. They concluded that HBeAg associates with IgG in both the circulation and kidney tissue. HBeAg is revealed as the antigen most likely responsible for the immunologic injury in HBV-associated MN [7].

**Figure 1:**
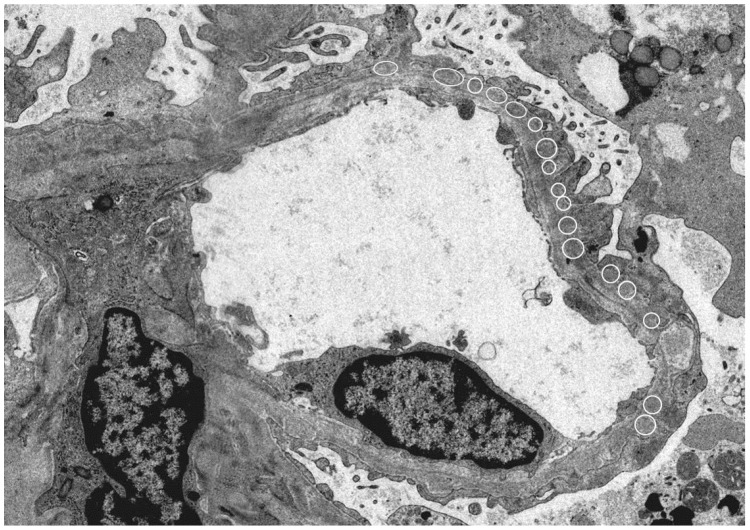
Electron microscope: Kidney histology of a patient with hepatitis B virus (HBV)-related membranous nephropathy (MN). There was irregularity of the basement membrane and a large number of granular accretions (open circle) were recognized on the basement membrane subcutaneously. (Image courtesy of Dr. Tadashi Yamamoto , Center of Biofluid Biomarker, Niigata University, Niigata, Japan).

Since HBe antigen incurs a positive electric charge, and the immunoglobulin G (IgG) immune complex has a low molecular weight, immune deposition occurs under the glomerular epithelium and has a negative electric charge when passing through the basement membrane (our case; Figure 1).

In addition, further immune complexes are formed to implant antigens; thus, some factors such as complement activation, platelet aggregation, polymorphonuclear leukocyte permeation, and the formation of fibrinous deposits are correlated and can lead to glomerular damage [8].

On the other hand, Xin et al. highlighted the impact and significance of HBV DNA in the pathogenesis of HBV-associated nephropathy. Renal tissue from cases with HBV-associated glomerulonephritis was examined for HBV DNA by in situ hybridization assay. HBV DNA was identified in most cases and was generally distributed in the nucleus and cytoplasm of the epithelial cells and mesangial cells of the glomeruli and in the epithelial cells of the renal tubules [9].

HBV-related MN has a favorable prognosis in children, with several high-prevalence areas reporting stable renal function and high rates of spontaneous remission. On the other hand, adults with HBV-related MN usually develop progressive disease because of a greater tendency than children to have hypertension, renal dysfunction, and clinical evidence of liver disease. Additionally, a national cohort study indicates that untreated chronic HBV infection is associated with an increased risk of end-stage of renal disease [10].

#### 2.1.2. Membranoproliferative Glomerulonephritis (MPGN)

MPGN associated with chronic HBV infection is characterized by the deposition of circulating antigen-antibody complexes in the mesangium and subendothelial space. These glomerular deposits consist mainly of IgG and C3, although their exact role remains uncertain [11].

#### 2.1.3. Polyarteritis Nodosa (PAN)

Renal involvement in hepatitis B-polyarteritis nodosa (HBV-PAN) usually occurs in the form of hypertension, microscopic hematuria, or proteinuria. Circulating antigen-antibody complexes aggregate in the vessels. The renal pathology may be limited to medium-sized arteries with ischemic changes in the glomeruli [12].

### 2.2. Treatment of HBV-Associated Nephropathy

In the treatment of HBV-related nephritis, it is important that the hepatitis B viral load is reduced since it is a causative antigen. Furthermore, the onset of HBV-related kidney disease is remarkably decreased by the spread of HBV vaccines. However, in cases of adult-onset HBV disease, the reported prognosis are poor [13]. Corticosteroid therapy has been used in some patients with HBV-associated nephropathy as a therapeutic trial for the symptomatic relief of proteinuria [14]. However, we should take notice of HBV reactivation, which is defined by an abrupt rise in HBV replication following immunosuppression [15].

This differs from childhood disease, in which there is a high rate of spontaneous remission [16]. In children, HBV-associated membranous nephropathy resolves spontaneously in many cases, usually in association with the appearance of free anti-HBeAb in the circulation [17]. Adults with HBV-related MN typically undergo a progressive disease course [18].

Currently, an ideal therapeutic agent has not been discovered. Conventional and pegylated interferon-α possess both immunoregulatory and antiviral effects [19]. IFN and NAs have different characteristics, but both were effective in terms of proteinuria remission and HBeAg clearance. Future, high-quality, large-scale RCTs should provide more reliable results for evidence-based medicine and the clinical drug treatment of HBV-MN patients [20].

## 3. Kidney Injury with the Hepatitis B Treatment Drug

The oral antiviral effect of NAs in CHB treatment is currently well tolerated, with minimal adverse effects. But long-term use of the NAs, are reported as extrahepatic effects, such as muscular disorders, bone disorders, neuropathies and renal dysfunction. Therefore, an important consideration when using nucleotide analogues is to dose-adjust according to the degree of renal impairment. Adefovir dipivoxil (ADV), which is a nucleotide analogues, these drugs are excreted in the urine following filtration in the glomerulus and renal tubule after they have been metabolized. Organic anion transporter (OAT) 1 is the mechanism for uptake to a renal tubule cell. In addition, multidrug-resistant protein (MRP) 4 and 2 participate in the discharge of these drugs from a renal tubule cell to a collecting duct. It is thought that a drug taken in by a renal tubule cell causes mitochondrial obstacles, and renal damage occurs. Renal functional decline that is frequently seen during chronic hepatitis B (CHB) treatment can influence the development of adverse effects, which affects overall prognosis [21].

Mitochondrial functional disorder of the proximal tubule cell was suspected due to the renal disorder caused by ADV. This Fanconi syndrome represents various syndromes associated with general functional disorders of the proximal tubule. This syndrome exhibits an increase in amino acid, glucose, uric acid, and bicarbonate acid levels, and is an obstacle to the resorption of phosphorus and the excretion of other urinary components [22].

Tenofovir disoproxil fumarate (TDF), which was superior to adefovir for nephropathy, was also developed as an anti-HIV drug. The effects of this drug on renal outcomes remain to be determined [23,24].

Moreover, Tenofovir alafenamide (TAF) is an oral, bioavailable prodrug of tenofovir that may have less renal and bone toxicity. It was noted that according to the World Health Organization (WHO) ARV consolidated guidelines, a baseline measurement of creatinine is not required for initiating ART along with the preferred tenofovir-based regimen in HIV-infected persons.

TAF is formulated to deliver the active metabolite to target cells more efficiently than TDF and at lower doses, thereby reducing systemic exposure to tenofovir. In patients with chronic hepatitis B, TAF appears to be as effective as TDF, with lower bone and renal toxicity. TAF has potential advantages in that dose adjustment is not required in patients with renal impairment, and monitoring can be less intense because of the better safety profile [25]. Renal and bone effects were significantly reduced in patients administered TAF [26].

## 4. Hepatitis B Cirrhosis and Renal Failure

Acute kidney injury (AKI) is common in patients with liver cirrhosis, including that caused by HBV, occurring in nearly 20% of patients with cirrhosis admitted to the hospital. In addition, chronic kidney disease (CKD) occurs in approximately 1% of all patients with cirrhosis [27]. If patients with chronic HBV did not suffer from HBV-associated nephropathy, the later stages of cirrhosis often caused renal dysfunction. This is a recognized as hepatorenal syndrome (HRS). The International Ascites Club (IAC) diagnostic criteria for hepatorenal syndromes state that HRS is a syndrome that occurs in patients with advanced liver disease, characterized by impaired renal function and marked abnormalities in the arterial circulation and over-activity of the endogenous vasoactive systems. In the kidney, there is marked renal vasoconstriction that results in a low GFR. In the extrarenal circulation, there is a predominance of arterial vasodilation that results in the reduction of systemic vascular resistance and arterial hypotension. HRS has been classified into two different clinical types: type-1 HRS, characterized by a rapidly progressive reduction of renal function, defined by a doubling of the serum creatinine to a level [2.5 mg/dL] in less than two weeks, and type-II HRS, in which the renal failure does not have a rapidly progressive course [28,29].

### 4.1. Mechanism of HRS

With the progression of cirrhosis, vasodilatation worsens and activated vasoconstrictive systems lead to renal vasoconstriction. Additionally, the increased cardiac output is now insufficient to maintain perfusion pressure (high-output heart failure) and further contributes to a decrease in renal blood flow and renal failure [27].

A member of the Acute Dialysis Quality Initiative (ADQI) and a member of the IAC proposed new criteria for cirrhosis and renal function failure in a working group in 2010. Type 1 liver renal syndrome and type 2 liver renal syndrome are included in the classification of CKD in the diagnostic criteria for AKI [30].

### 4.2. Treatment of HRS

Generally, prevention is important, and antibiotics, non-steroidal anti-inflammatory drugs, and contrast media which cause nephrotoxicity should be used carefully. In addition, we should be careful about inducing excessive diarrhea using lactulose for hepatic encephalopathy and renal ischemia caused by large-volume paracentesis [31]. Terlipressin is widely used and α-adrenergic drugs have been used as a potential alternative. Among α-adrenergic drugs, midodrine given orally together with octreotide given subcutaneously or norepinephrine given by continuous intravenous infusion has been used. Recently, terlipressin was shown to be superior to midodrine plus octreotide in the treatment of type-1 HRS [32]. Treatment options are the administration of these pharmacological treatments with albumin and placement of transjugular intrahepatic portosystemic shunts, but these treatments do not improve the survival rate. Liver transplantation is the definitive treatment for patients with HRS.

## 5. Equivalent Renal Failure in Liver Cirrhosis

The liver is the major organ for the metabolism of the major nutrients: proteins, fats, and carbohydrates. Liver cirrhosis (LC) is often complicated by protein-energy malnutrition and sarcopenia, which are linked to unfavorable clinical outcomes in patients with LC. The number of patients with LC with decreased serum creatinine levels and eGFR renal function may be overestimated [33]. Therefore, it is very important to detect renal failure in its early stages before it progresses to end-stage renal disease.

Serum cystatin C should be measured as a complementary test to serum Cr when renal function is assessed in patients with cirrhosis, particularly in women and in those with sarcopenia [34]. The biomarkers used for detection of renal failure are albumin, serum creatinine, and serum urea nitrogen, which can be tested from urine samples. The biomarker which can predict AKI earlier than serum creatinine and volume of urine is often identified. Cystatin C is a protein with a molecular weight of 13 kDa. This low molecular-weight protein is secreted by existing nuclear cells of the whole body. After it is filtered in the glomerulus, most of it is reabsorbed in the proximal tubule. It is not affected by the number of muscles, sex, age, exercise, or diet, and it is more useful than serum creatinine.

In the past few years, new plural biomarkers were developed, and they are promising from our point of view. Among the biomarkers established in a large-scale cohort study, neutrophil gelatinase-associated lipocalin (NGAL), interleukin-18 (IL-18), 1 kidney injury molecule (KIM-1), liver fatty acid-binding protein (L-FABP), 2 tissue inhibitor metalloproteinase inhibitor (TIMP-2), and 7 insulin growth factor-binding protein (IGFBP7) are currently noted (Table 1).

## 6. Conclusions

Many liver diseases, including the liver diseases derived from hepatitis B, are related to many organs and other diseases, including the hepatorenal syndrome. It is necessary to try to detect and examine early renal dysfunction using the new biomarkers.

## Figures and Tables

**Table 1:** Useful biomarkers for renal dysfunction in liver cirrhosis.

Biomarker	Pathophysiological Process	Authors	Characteristic
Cystatin C	Kidney function (GFR)	Mindikoglu, A.L. et al. [34]	Serum cystatin C avoids the limitations of glomerular filtration rate related to the patients with cirrhosis, particularly in women and those with sarcopenia.
Neutrophil gelatinase-associated lipocalin (NGAL)	Tubulointerstitial injury	Yoshikawa, K. et al. [35]	NGAL, a ubiquitous lipocalin iron-carrying protein, 45 is highly expressed in the tubular epithelium of the distal nephron and released from tubular epithelial cells following damage such as AKI.
Tissue inhibitor of metalloproteinases-2 (TIMP-2)	Inflammation	Horstrup, J.H. et al. [36]	Urinary concentrations of tenascin and TIMP-1 are elevated in association with renal disease and may reflect specific aspects of renal fibrosis.
Kidney injury molecule-1 (KIM-1)	Tubulointerstitial injury	Waanders, F. et al. [37]	KIM-1 is a transmembrane tubular protein with uncertain function, not detectable in the normal kidney, but elevated in experimental and clinical kidney damage.
IGF-binding protein-7 (IGFBP7)	Inflammation	Aregger, F. et al. [38]	The protein has been implicated in these processes it is believed editing might affect apoptosis, regulation of cell growth and angiogenesis.
Liver-type fatty acid binding protein (L-FABP)	Tubulointerstitial injury	Kamijo, A. et al. [39]	L-FABP is expressed in proximal tubular cells and is a biomarker of inflammation investigated in diabetes, diabetic nephropathy, hypertension, and early CKD.
Interleukin-18 (IL-18)	Inflammation	Chirag R. Parikh et al. [40]	IL-18 was markedly increased in patients with established AKI from ischemia, but not in patients with AKI from urinary tract infection, CKD, nephrotic syndrome, or those with prerenal failure.
